# Identifying Supports and Barriers to Physical Activity in Patients at Risk for Diabetes

**Published:** 2006-09-15

**Authors:** Katrina E Donahue, Thelma J Mielenz, Leigh F Callahan, Philip D Sloane, Robert F Devellis

**Affiliations:** University of North Carolina at Chapel Hill, Department of Family Medicine; Cecil G. Sheps Center for Health Services Research and Thurston Center for Arthritis Research, Chapel Hill, NC; Cecil G. Sheps Center for Health Services Research and Thurston Center for Arthritis Research, Chapel Hill, NC; University of North Carolina Department of Family Medicine and Cecil G. Sheps Center for Health Services Research, Chapel Hill, NC; Thurston Center for Arthritis Research, Chapel Hill, NC

## Abstract

**Introduction:**

Recent clinical trials have demonstrated that increasing physical activity among patients at risk for diabetes can prevent or delay the onset of type 2 diabetes. In this study, we surveyed primary care patients at risk for diabetes to 1) describe physical activity habits, supports, and barriers; 2) identify characteristics associated with increased physical activity; and 3) develop and assess the psychometric properties of an instrument that measures influences on physical activity.

**Methods:**

A cross-sectional sample of 522 high-risk adults who attended 14 North Carolina primary care family practices were mailed a survey about physical activity and supports of and barriers to physical activity. Risk status was determined by the American Diabetes Association's diabetes risk test. Exploratory principal components factor analyses were conducted on the influences on physical activity instrument. Predictive logistic regression models were used for the dichotomous outcome, meeting recommended *Healthy People 2010* activity levels.

**Results:**

Of the 258 respondents (56% response rate), 56% reported at least 150 minutes of moderate or vigorous activity per week. Higher education remained a significant demographic predictor of activity (odds ratio [OR], 1.72; 95% confidence interval [CI], 1.08–2.75). Participants were less likely to be physically active if they reported that activity is a low priority (OR, 0.45; 95% CI, 0.23–0.89), were worried about injury (OR, 0.42; 95% CI, 0.25–0.69), or had difficulty finding time for activity (OR, 0.38; 95% CI, 0.17–0.87).

**Conclusion:**

Participants at risk for diabetes who prioritize physical activity, make time for activity, and are less worried about injury have higher odds of being physically active. Primary care practice and community interventions should consider targeting these areas of success to increase physical activity in sedentary individuals at risk for diabetes.

## Introduction

Physical inactivity contributes to the increasing prevalence of diabetes, a disease that affects 20 million individuals in the United States (1). A similarly large number of individuals have prediabetes and are at high risk for progression to diabetes. Current estimates are that more than 40 million individuals in the United States have prediabetes (1). In an effort to combat this trend, three recent clinical trials of exercise interventions in patients with impaired glucose tolerance reduced the risk of developing diabetes over 4 to 6 years by 46% to 58% (2-4). Translating these results to the general population could have a large impact on reducing the incidence of diabetes. Primary care physicians' offices, which see many patients with diabetes and prediabetes, are a promising site for physical activity promotion.

Initiating and maintaining regular physical activity remain difficult challenges (5,6), and more research is needed on the factors that motivate people at risk for diabetes to engage in physical activity. Studies examining why people are nonadherent to exercise in general point to three groups of obstacles: individual influences, social influences, and environmental influences. Individual influences include lack of interest, low priority for health promotion, depressive symptoms, physical limitations, smoking, and female sex (7-10). Social influences include lack of social support from friends and family (8,9). Environmental influences include weather and accessibility and availability of places for activity (7,11). Individuals at risk for diabetes would particularly benefit from increasing physical activity. More research is needed about which of these obstacles to activity are more prominent in this group.

In addition to potential barriers to activity, research on physical activity needs to identify facilitating factors, such as support, that promote success. For example, health professionals, who daily encounter patients who are at risk for diabetes, may play an important role; having a physician discuss physical activity with patients has been found to be a predictor of engaging in activity (12). However, only 20% to 35% of health care providers report assessing physical activity, and only 11% give written exercise plans to their patients (12-14). Obtaining information about supports of physical activity in patients at risk for diabetes could be helpful with patient counseling.

In this study, we surveyed primary care patients at risk for diabetes to 1) describe physical activity habits, supports, and barriers (including demographics; self-efficacy; stage of change; and individual, social, and environmental influences on physical activity); 2) identify which characteristics are associated with increased activity in this sample; and 3) develop and assess the psychometric properties of an instrument that measures influences on physical activity.

This study attempts to look at which influences on physical activity are predominant in patients at risk for diabetes. Information on physical activity behavior, supports, and barriers are important for tailoring physical activity interventions in this group.

## Methods

### Design overview

In this cross-sectional study, a subsample of participants at high risk for diabetes in the North Carolina Health Project (NCHP) cohort was mailed a survey about physical activity, supports, and barriers. NCHP participants were recruited from the North Carolina Family Medicine Research Network (NCFMRN) (15). The purpose of the NCHP was to develop a representative cohort of adult primary care patients for use in multiple projects. Details of the development of the cohort are described elsewhere (15). This study was reviewed and approved by the University of North Carolina Biomedical Institutional Review Board.

### Population 

The NCHP cohort was established in spring and summer 2001. A total of 4876 individuals aged 18 years and older from the NCFMRN family practice sites completed a baseline  enrollment questionnaire that included questions about health-related quality of life (16,17), comorbid conditions, and risk factors for diabetes (18). In each practice office, all adults seen during a 1-month enrollment period were offered the opportunity to participate; the enrollment rate was 62%. The mean age of the NCHP cohort at baseline was 47.7 years; 70.8% were female, and 19.7% were African American (15).

Participants in the NCHP cohort were determined to be at risk for diabetes according to their original responses to the American Diabetes Association (ADA) diabetes risk test questions (18). These questions included age, body mass index (BMI), family history of diabetes, history of gestational diabetes, and exercise ("I get little or no exercise in a typical day"). A total score of 10 points or higher placed a person at high risk. Sensitivities of the diabetes risk test have been described as 69% to 78%, and specificities for prediabetes or diabetes are 51% to 54% (19). In the original NCHP cohort, 45% of the adults reported performing little or no exercise in a typical day; 15% reported having diabetes; and an additional 36% scored 10 or higher (high risk) on the ADA test. In comparison, 6.5% of people in North Carolina reported having diabetes in the Behavioral Risk Factor Surveillance System (BRFSS) (20). 

### Sampling

Of the 4876 people who completed the NCHP baseline questionnaire, 4139 (85%) consented to receive additional surveys. The two groups, those who consented to receive further surveys and those who did not, differed in age (mean age 47.7 years for those who consented compared with 43.7 years for those who did not, *P* < .001), BMI (mean BMI 29.5 kg/m^2^ for those who consented compared with 28.1 kg/m^2^ for those who did not, *P* < .001), and having less than a high school education (20.5% for those who consented compared with 13.5% for those who did not, *P* < .001), but they did not differ by sex or race. Of the 4876 baseline enrollees, there were 1077 people at risk for diabetes who 1) did not currently have diabetes, 2) were white or African American, 3) consented to receive surveys, and 4) had a valid mailing address. This group included 204 white men, 627 white women, 193 African American women, and 53 African American men. In April 2003, we mailed a self-report questionnaire assessing physical activity supports and barriers to a random stratified sample of 522 adults of the 1077 adults identified by the 2001 enrollment questionnaire as being at high risk for diabetes. These 1077 adults were stratified by race and sex to assure adequate representation in our sample. The initial mailing was accompanied by a self-addressed, stamped return envelope, and a postcard reminder was mailed 2 weeks later. Nonrespondents were mailed a second questionnaire 1 month later. Attempts were made to telephone individuals who did not respond, and they were asked to complete the survey over the telephone. 

### Variables collected

In addition to baseline demographic data obtained from the original NCHP cohort survey, the survey designed for this study included physical activity items adapted from the BRFSS (converted from telephone to written survey) (21). The physical activity outcome for this study, specified as an objective in *Healthy People 2010* (22), is 150 minutes or more of moderate to vigorous activity per week (calculated by the responses to the BRFSS activity questions). A checklist of physical activities respondents enjoyed (walking, running, biking, stretching, water aerobics, gardening, tennis, swimming, lifting weights, yoga, hiking, other) and whether or not their health provider talked with them about physical activity in the past year were also included in the survey. Questions also addressed stage of change for physical activity (23), whether they had to cut back on activity because of health in the past 6 months, and self-efficacy using the Self-Efficacy for Exercise (SEE) scale (24). We also included items on influences on physical activity in an assessment instrument, the Influences on Physical Activity Instrument (IPAI).

### Instrument development 

The IPAI was included as part of the mailed survey to assess physical activity barriers and supports based on factors reported in the literature to be associated with activity (7-12,25,26) as well as questions included in the "Starting the Conversation" tool for physical activity (used by many doctors offices in North Carolina) (27). The IPAI consisted of three separate domains, each representing a different type of influence — individual, support, and environment. (The IPAI and information about its scoring is available in the Appendix.) The IPAI is written for a fourth- to sixth-grade reading level. Comprehension was assessed qualitatively through written and oral feedback by 15 white and African American adults (six were older than 65 years, and nine were younger than 65 years) at the University of North Carolina at Chapel Hill Family Practice before survey administration.

### Analysis

All statistical analyses were performed using Stata (StataCorp, College Station, Tex). Univariate and bivariate analyses of the continuous and categorical variables were performed for missing data, sparse numbers, extreme values, and linearity. Bivariate analyses evaluated the unadjusted relationship between the covariates and the physical activity outcome of 150 minutes or more of moderate to vigorous activity per week.

Exploratory principal components factor analysis (PCFA) were conducted on each of the three domains of the IPAI (individual, support, and environmental influences) to clarify the factor structure of each domain. Kaiser's eigenvalue greater-than-1 rule and Cattell's scree test were used to determine the number of factors to extract (28,29). An eigenvalue equal to 1.0 meant that a component accounted for the average variance of one item (28). A scree test included a plot of the eigenvalues on the *y* axis and consecutive factors on the *x* axis, and the last factor retained was before the downward curve where the slope levels off (29). If more than one factor was present, then the factors were rotated using an oblique rotation to determine if each item would load on a single factor. The Cronbach α reliability coefficient was calculated after the PCFA for each factor present.

Predictive logistic regression models were used for the dichotomous outcome, reaching 150 minutes or more of moderate to vigorous activity per week. Independent variables, including the IPAI and SEE scale, were assessed as continuous variables in the model. Education was assessed in three categories: some high school or less, high school graduate, and some college or more, with some high school or less as the reference category. All other variables were dichotomized; age was dichotomized into younger than 55 years and 55 years and older after testing for the linearity assumption. Obesity was defined as having a BMI of 30 kg/m^2^ or higher. Interaction terms, sex with age and race with all other covariates, were evaluated during exploratory analyses to help identify which risk factors may be effect modifiers. A backwards stepwise variable reduction was used to identify important variables. Interaction terms, race with age and race with working status, were significant in the exploratory analyses but were dropped from the final model by a *P* value of .05 for the −2 Log likelihood ratio test. The covariates including age, sex, race, and work status were also dropped from the logistic model using the −2 Log likelihood ratio test. For the final model, a population average effects model was used to adjust for any intraclass correlation due to the cluster sampling design. Clusters were the practice sites in this analysis.

## Results

Of the 522 mailed surveys, 30 had inaccurate or incomplete addresses, and 30 had telephone numbers that were no longer in service. Of the remaining 462, 258 surveys were completed, for a response rate of 56% (180 returned after first mailing and postcard, 31 returned after second mailing, and 47 completed with telephone call). In comparison with respondents, nonrespondents tended to be younger (mean age 49 years for nonrespondents vs 54 years for respondents, *P* < .001) and single (47.9% for nonrespondents vs 38.4% for respondents, *P* = .03). There were no significant differences for sex, race, BMI, smoking status, education, rural residence, or work status between respondents and nonrespondents. 

Baseline characteristics of the respondents appear in Table 1; the average age was 54 years, and the majority were obese (mean BMI, 33.1 kg/m^2^). Slightly more than half (55.8%) reported meeting *Healthy People 2010* activity levels (i.e., 150 minutes or more of moderate to vigorous activity per week). The most frequently reported enjoyable activities were walking (70.2%), gardening (39.3%), stretching (23.7%), swimming (12.2%) and biking (10.7%) (data not shown). In the past year, 69% of this high-risk group had discussed physical activity with their health care provider. Twenty-one percent of respondents were in the contemplation stage of change for physical activity. Half of the group noted reducing activity in the previous 6 months because of health.

### IPAI

**Figure 1:**
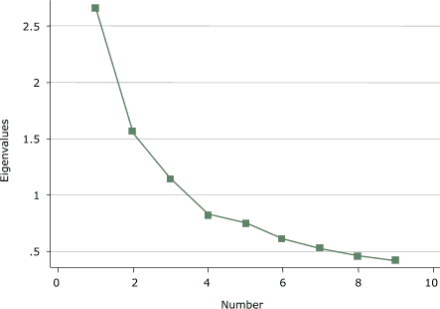
Scree plot of *individual influences* domain of the Influences on Physical Activity Instrument (IPAI) from a principal components factor analysis.

Exploratory PCFA of the *individual influences* domain of the IPAI identified three factors that explained 60% of the total variance among items (Figure 1). After an oblique rotation, the factor loadings made the interpretation of the three factors clearer. Four items (Appendix) loaded on factor 1 (items *a,* *c*, *d*, and *g*) and appeared to represent the view that physical activity is a low priority; two items loaded on factor 2 (items *e *and *f*) and appeared to represent weight control; and three items (items *h*, *j*, and *k*) loaded on factor 3 and appeared to represent injury concerns. Items *b* and *i* were dropped because they had loadings greater than 0.3 on more than one factor. For each item retained, the primary loading was greater than 0.58, and the secondary and tertiary loadings were never greater than 0.25. Thus, three scales comprise the *individual influences* domain of the IPAI.

**Table:** 

An average score was calculated for each of these three scales based on the factor structure. The items loading on factor 1 (low priority) had a Cronbach α of 0.68 and a mean score of 2.31; factor 2 (weight control) items had a Cronbach α of 0.67 and a mean score of 2.28; and factor 3 (injury concerns) items had a Cronbach α of 0.63 and a mean score of 2.22.

**Figure 2:**
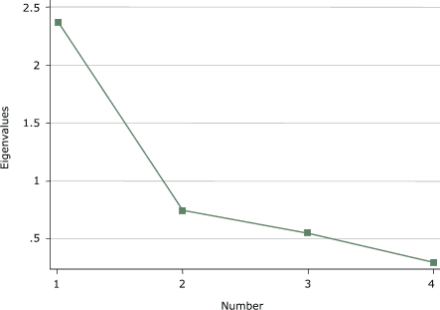
Scree plot of *support influences *domain of the Influences on Physical Activity Instrument (IPAI) from a principal components factor analysis.

Exploratory PCFA of the *support influences* domain identified one factor that explained 60% of the total variance among the items (Figure 2). Item *p *was dropped because it loaded weakly on one factor, and when two factors were forced, it loaded ambiguously. The factor loading for items *l *to *o *were all greater than 0.72. An average score was calculated for the support influences scale. The scale comprising the support items *l *to *o* had a Cronbach α of 0.77 and a mean of 2.28. 

**Table:** 

**Figure 3:**
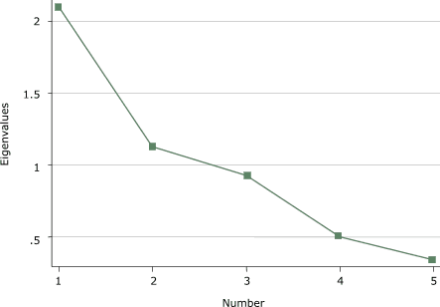
Scree plot of *environmental influences* domain of the Influences on Physical Activity Instrument (IPAI) from a principal components factor analysis.

The exploratory PCFA on the *environmental influences* identified two factors that explained 65% of the total variance among the items (Figure 3). The factor loadings became clearer after an oblique rotation. Three items (*q*, *r*, and *s*) appeared to represent having a place for activity (factor 1). Two items (*t* and *u*) appeared to represent having time for activity (factor 2). For each item on the two environmental influences scales, the primary loading was greater than 0.27, and the secondary loadings were never greater than 0.17. Factor 1 (having a place for physical activity) had a Cronbach α of 0.60 and a mean score of 2.13, and factor 2 (having time for physical activity) had a Cronbach α of 0.53 and a mean score of 2.23. 

**Table:** 

Interfactor correlations and a higher order factor analysis suggested that a global summary score based on the six scales was not the best way to represent IPAI. Therefore, the average score for the items loading on each factor was calculated and used in the modeling rather than a single summary score based on combining the six scales of the IPAI. 

### Predictors of activity

There were no significant differences between the characteristics of those who were physically active and those who were not, except for level of education (Table 2). A higher proportion of respondents with a college education or high school diploma (65.9%) reported meeting *Healthy People 2010* activity levels than of those with less than a high school diploma (30.9%), *P* < .001. SEE was higher among active individuals than inactive individuals (5.6 for active compared with 4.9 for inactive, *P* = .007), as were scores on several questions about influences on activity. Fewer physically active individuals reported that activity was a low priority (*P* < .001), disagreed that physical activity influences their weight (*P* = .02), and worried about injury (*P* < .001). Additionally, the active group less often had difficulty finding a place for activity (*P* < .001) and making time for activity (*P* < .001). Interestingly, health care provider counseling about physical activity was similar between groups. Social support was also similar.

In the multivariate model (Table 3), higher education remained a significant predicator of physical activity (OR, 1.72; 95% CI, 1.08–2.75). Participants who reported that physical activity was a low priority were less likely to be physically active than participants who reported that physical activity was a high priority (OR, 0.45; 95% CI, 0.23–0.89). Participants who reported concerns about injury were also less likely to be physically active (OR, 0.42; 95% CI, 0.25–0.69). Additionally, those who had difficulty finding time for activity were less likely to be physically active (OR, 0.38; 95% CI, 0.17–0.87). Beliefs about weight being affected by activity, having support for activity, having a place for activity, and self-efficacy were not significant independent predictors of physical activity in this sample.

## Discussion

We examined supports of and barriers to physical activity in people who visited the primary care practices of the NCFMRN. This study also includes the first use of the IPAI. We found that half of adult primary care patients at high risk for diabetes in these practices report meeting *Healthy People 2010* objectives for activity. Participants differed in several potentially modifiable characteristics as a function of reported activity level. Participants at risk for diabetes who place physical activity high on their priority list, make time for physical activity, and have fewer concerns about injury from physical activity seem to be more active.

Participants with a college education or high school diploma are more physically active than those without a high school diploma. Although not specifically examined in this study, education may reflect the types of jobs or the lack of time that individuals with less education may have. Lower education may also influence the understanding of the importance of physical activity. Education may be a marker for thinking about how to intervene in this group differently.

The IPAI assesses three domains using six multi-item scales containing two to five items each. This first version of the instrument yielded only fair Cronbach α values ranging from 0.53 to 0.77. Although the item groupings are probably more reliable than single items, the poor reliabilities reduce the opportunities for the variable in question to be a significant predictor. Further development of the IPAI will include adding items to the existing six scales to improve internal consistency and testing for construct validity. At this point, the six multi-item scales should be used as individual scales covering a domain of interest. These simple measures may decrease barriers to measuring influences on physical activity in the clinical setting, but further development of the whole IPAI is needed.

Other variables, including BMI, smoking, age, and race did not differ between the activity groups. This is most likely due to the homogeneity of the prediabetes population. These potential markers for activity are also risk factors for prediabetes. Scores on the SEE dropped out of the model in this population after adjusting for other variables. Additionally, there was no difference in rates of health care providers talking about physical activity with patients in the past year. Thus, although discussion of physical activity may still be beneficial, it may not be sufficient. In the Activity Counseling Trial (30), intensive counseling interventions for physical activity in primary care settings were more successful than brief advice for women, but this was not the case for men. Social support, although not significantly different between the active and inactive group in this study, may still be significant for certain subpopulations.

Findings from this study provide useful clues about potential barriers to and facilitators of physical activity in this population of people at risk for developing diabetes. Programs that encourage lifestyle interventions, in which activity is accumulated during the day instead of structured into a gym workout, have been examined in sedentary populations and are possible methods of intervention for this high-risk group (31,32). In addition, education targeted at reducing concerns about injury by including ways to reduce injuries may be particularly important in this group; more than half of the participants had to reduce their physical activity because of health problems in the past 6 months.

Limitations of this study include that it is based on self-reported data and is cross-sectional in design; there is no way to tell if the differences between the active and inactive groups are causal or merely an association. Additionally, individuals may overestimate their activity levels. The survey response rate was moderate but similar to that of the BRFSS (33). Literacy may also have been an issue; 21.3% of respondents reported an education level of some high school or less. Some participants may have had difficulties understanding previously validated scales such as the SEE (24), which was noted by some participants during pilot testing.

Patients at risk for developing diabetes who prioritize physical activity, make time for activity, and are less concerned about injury have higher odds of being physically active. Given these observed differences between active and inactive people, primary care practice and community interventions should target these findings in this rapidly growing population. Increasing activity in this high-risk group may have a significant effect on diabetes prevention.

## Figures and Tables

**Table 1:** Characteristics of Physical Activity Survey Participants (n = 258*), *North Carolina Health Project, 2003

**Characteristic**	**Value**
Mean age, y (SD)	54.1 (14.0)
Female, %	60.5
African American, %	40.0
Education level, %	
Some high school or less	21.3
High school graduate	28.7
Some college or more	50.0
Married, %	61.6
Rural, %	61.8
Ever smoked, %	50.7
Mean body mass index, kg/m^2^ (SD)	33.1 (7.6)
Type of work, %	
Not employed	50.6
Sitting or standing	29.2
Walking or heavy labor	20.2
Meeting *HP 2010* physical activity objectives, %	55.8
Stage of change, %	
Precontemplation	16.1
Contemplation	21.0
Preparation	23.0
Action	9.7
Maintenance	30.2
Reduced activity because of health (last 6 months), %	50.4
Health care provider talked about physical activity (in past year), %	69.0

*HP 2010* indicates *Healthy People 2010*.

**Table 2:** Comparisons Between Inactive and Active Survey Participants (n = 258), North Carolina Health Project, 2003^a^

**Characteristic**	**Inactive (n = 114 )**	**Active (n = 144)**	***P* Value**
Age, y			
18-39	50.0	50.0	.15
40-64	39.7	60.3	
≥65	53.4	46.5	
Female	51.9	48.1	.12
Race			
African American	50.5	49.5	.10
White	40.0	60.0	
Education			
Some high school or less	69.1	30.9	<.001
High school graduate	43.2	56.8	
Some college or more	34.1	65.9	
Married	39.6	60.4	.06
Rural	45.4	54.6	.32
Smoker	49.1	50.9	.25
Health provider talked about physical activity in past year	64.9	72.3	.20
Self-efficacy score on Self-Efficacy for Exercise scale, mean (SD)	4.9 (2.1)	5.6 (1.8)	.007
**Influences on Physical Activity Instrument (IPAI), mean (SD)**			
Low priority	2.5 (0.55)	2.1 (0.49)	<.001
No weight benefit	2.4 (0.60)	2.2 (0.59)	.02
Injury concerns	2.4 (0.53)	2.1 (0.48)	<.001
Little support	2.3 (0.53)	2.2 (0.56)	.06
No place for activity	2.3 (0.51)	2.0 (0.49)	<.001
No time for activity	2.4 (0.59)	2.1 (0.49)	<.001

a Values are percentages unless otherwise indicated.

**Table 3:** Logistic Regression of Factors Associated With Physical Activity in Patients at Risk for Diabetes, North Carolina Health Project, 2003^a^

**Characteristic**	**Odds Ratio (95% CI)**	***P* Value**
Higher education	1.72 (1.08-2.75)	.02
Married	1.73 (0.90-3.34)	.10
Obese	1.72 (0.80-3.72)	.17
Health care provider talked about physical activity	1.64 (0.95-2.83)	.08
Self-efficacy for exercise	1.05 (0.91-1.22)	.47
**Influences on physical activity**		
Individual		
Low priority	0.45 (0.23-0.89)	.02
No weight benefit	0.87 (0.50-1.50)	.63
Injury concerns	0.42 (0.25-0.69)	.001
Support		
Little support	0.82 (0.34-1.97)	.66
Environmental		
No place for activity	0.91 (0.38-2.18)	.84
No time for activity	0.38 (0.17-0.87)	.02

CI indicates confidence interval.

a Adjusted for cluster design.

## APPENDIX. Influences on Physical Activity Instrument (IPAI)

Below are some statements about physical activities and exercise. To the right of each statement, check the ONE box that best describes whether you strongly agree, agree, disagree, or strongly disagree.

**Table:**

**1. Individual**	**Strongly Agree**	**Agree**	**Disagree**	**Strongly Disagree**
**a.** I find it hard to work up the energy for physical activity.^a^	□_1_	□_2_	□_3_	□_4_
**b.** *Physical activity increases my energy.*	□_1_	□_2_	□_3_	□_4_
**c.** Physical activity is low on the list of things I want to do.^a^	□_1_	□_2_	□_3_	□_4_
**d.** Most physical activity is just plain boring.^a^	□_1_	□_2_	□_3_	□_4_
**e.** Physical activity helps me lose weight.^b^	□_1_	□_2_	□_3_	□_4_
**f.** Physical activity helps me eat less.^b^	□_1_	□_2_	□_3_	□_4_
**g.** I'm overweight, so it's hard to find ways to be active.^a^	□_1_	□_2_	□_3_	□_4_
**h.** I worry about "overdoing it" when I exercise.^c^	□_1_	□_2_	□_3_	□_4_
**i.** * It feels good to exercise.*	□_1_	□_2_	□_3_	□_4_
**j.** I'm worried that exercise may do more harm than good.^c^	□_1_	□_2_	□_3_	□_4_
**k.** Physical activity makes me sore and uncomfortable.^c^	□_1_	□_2_	□_3_	□_4_

Note: Italic items were dropped.

a Factor 1: low priority.

b Factor 2: weight control.

c Factor 3: injury concerns.

**Table:**

**2. Support**	**Strongly Agree**	**Agree**	**Disagree**	**Strongly Disagree**
**l.** I have friends who are physically active.^a^	□_1_	□_2_	□_3_	□_4_
**m.** My friends will do physical activity with me.^a^	□_1_	□_2_	□_3_	□_4_
**n.** I have family members who are physically active.^a^	□_1_	□_2_	□_3_	□_4_
**o.** My family will do physical activity with me.^a^	□_1_	□_2_	□_3_	□_4_
**p.** *I talk with my health care provider about physical activity.*	□_1_	□_2_	□_3_	□_4_

Note: Italic item was dropped.

a Factor 1: support.

**Table:**

** 3. Environmental**	**Strongly Agree**	**Agree**	**Disagree**	**Strongly Disagree**
**q.** I have a place to do physical activity.^a^	□_1_	□_2_	□_3_	□_4_
**r. **There are places that are easy to get to so I can do physical activity.^a^	□_1_	□_2_	□_3_	□_4_
**s.** The activities I want to do cost too much.	□_1_	□_2_	□_3_	□_4_
**t.** I can make time for physical activity.^b^	□_1_	□_2_	□_3_	□_4_
**u.** It is hard to find time to be physically active.^b^	□_1_	□_2_	□_3_	□_4_

a Factor 1: place for activity.

b Factor 2: time for activity.

**Scoring for IPAI**

The IPAI, version 1, assesses three domains of influence on physical activity using six multi-item scales that contain two to five items each. The *individual influences* domain contains three scales (low priority, weight control, and injury concerns). The *support influences* domain identified one support scale, and the *environmental influences* domain identified two scales (place for activity and time for activity). This first version yielded Cronbach α values ranging from 0.53 to 0.77.

To compute the score for each IPAI subscale, add the indicated value for each question, reverse score (R) any negative statements, and calculate the mean value.

**Individual domain**

Low priority scale = a(R) + c(R) + d(R) + g(R)/ 4

Weight control scale = e + f/ 2

Injury concerns scale = h(R) + j(R) + k(R)/ 3

**Support domain**

Little support scale = l + m + n + o/4

**Environmental domain**

No place for activity scale = q + r + s(R)/3

No time for activity scale = t + u(R)/2
