# Erratum, Vol. 03, October 2006 Release

**DOI:** 10.5888/pcd10.060011e

**Published:** 2013-05-09

In the article “Identifying Supports and Barriers to Physical Activity in Patients at Risk for Diabetes,” formulas for calculating the mean of scales were incorrect. The formulas appear in the Appendix under the heading “Scoring for IPAI.” Brackets were omitted for the numerators.

In the individual domain, the corrected formulae are the following:

Low priority scale = [a(R) + c(R) + d(R) + g(R)]/ 4

Weight control scale = [e + f]/2

Injury concerns scale = [h(R) + j(R) + k(R)]/3

In the support domain, the corrected formula is “Little support scale = [l +m +n+o]/4.”

In the environmental domain, the corrected formulae are “No place for activity scale = [q + r + s(R)]/3” and “No time for activity scale = [t + u(R)]/2.”

Corrections were made to our website on April 29, 2013, and appears online at http://www.cdc.gov/pcd/issues/2006/oct/06_0011.htm. We regret any confusion or inconvenience this error may have caused.

